# Case report: Cardiac intimal sarcoma in a young child

**DOI:** 10.3389/fped.2023.1238847

**Published:** 2023-09-25

**Authors:** Sanne Verbeek, Raf Sciot, Maria Debiec-Rychter, Veerle Labarque, Bart Meyns, Bjorn Cools

**Affiliations:** ^1^ Department of Pathology, University Hospitals Leuven, Leuven, Belgium; ^2^ Center for Human Genetics, Catholic University Leuven, Leuven, Belgium; ^3^ Department of Pediatric Hematology and Oncology, University Hospitals Leuven, Leuven, Belgium; ^4^ Department of Cardiac Surgery, University Hospitals Leuven, Leuven, Belgium; ^5^ Department of Pediatric and Congenital Cardiology, University Hospitals Leuven, Leuven, Belgium

**Keywords:** intimal sarcoma, cardiac, child, MDM2, right ventricle (RV)

## Abstract

Undifferentiated mesenchymal tumors from the intimal layer (intimal sarcomas) are rare within the ventricles and exceptional in children. A rare case of an intimal sarcoma located in the right ventricle in a young child is presented with need for urgent surgical resection due to mechanical flow obstruction. Tumor cells showed amplification of *MDM2* gene and a homozygous loss of *CDKN2A* on 9p21. A review of the literature regarding primary cardiac malignancies and intimal sarcoma in children is provided.

## Introduction

Cardiac intimal sarcomas are very rare. In contrast to vascular intimal sarcoma, cardiac involvement is an infrequent finding especially in children. A retrospective study of 100 cardiac sarcomas however, describes intimal sarcoma as the predominant primary sarcoma of the heart (1). These tumors are known to be very aggressive with high metastatic potential and often (micro-)metastatic disease at the time of diagnosis. Prognosis is therefore reserved with full surgical resection as the major outcome parameter for prolonged survival (2–4).

We present a case of a 4-year old child presenting with an intimal sarcoma located in the right ventricle with need for urgent surgical resection. This is a one of the few reports of cardiac intimal sarcoma and the first report of a ventricular localization in a young child.

## Case description

A 4-year old child was admitted to the pediatric cardiology department with suspicion of right heart failure: ascites, edema and poor circulation. There was a history of progressive fatigue and exertional dyspnea for some days. Medical history was negative apart from food allergy.

The patient presented with edema of the lower limbs with poor circulation, abdominal distention with hepatomegaly. There was no cardiac murmur, heart rate was 53 BPM, blood pressure of 101/73 mmHg and oxygen saturation of 100%.

**Figure 1:**
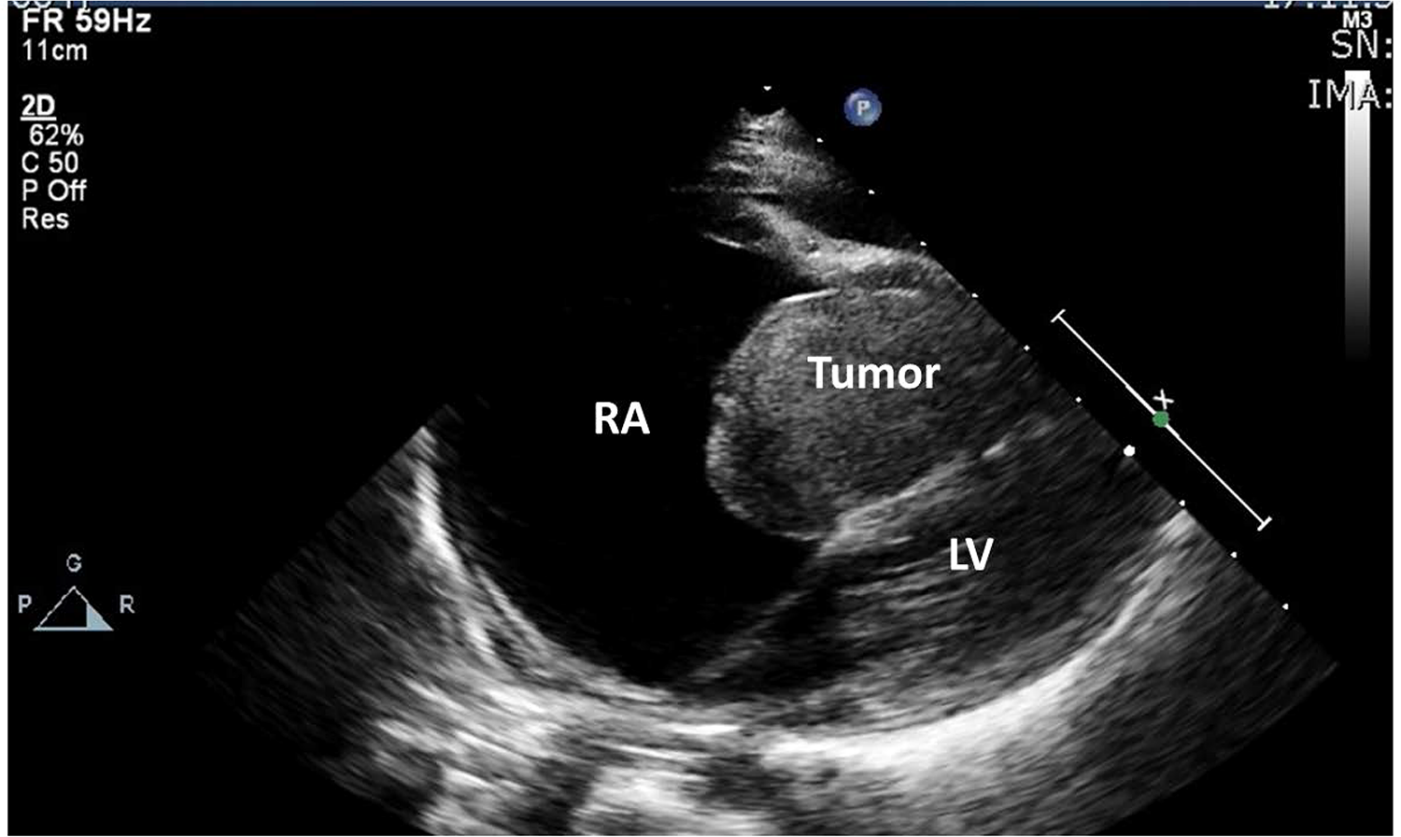
Echocardiographic image of large dilated right atrium with large mass in the right ventricular cavity towards the right atrium; no discernable tricuspid valve.

Abdominal ultrasound confirmed ascites and hepatomegaly. Chest x-ray showed cardiomegaly with presence of bilateral pleural effusion. The cardiac ultrasound revealed a large mass in the right ventricle bulging through tricuspid valve into the right atrium; delineation of the leaflets of the tricuspid valve was impossible due to the mass. (Figure 1) The right atrium was importantly dilated. No regurgitation of the tricuspid or pulmonary valve could be observed. Pericardial and pleural effusion were confirmed.

**Figure 2:**
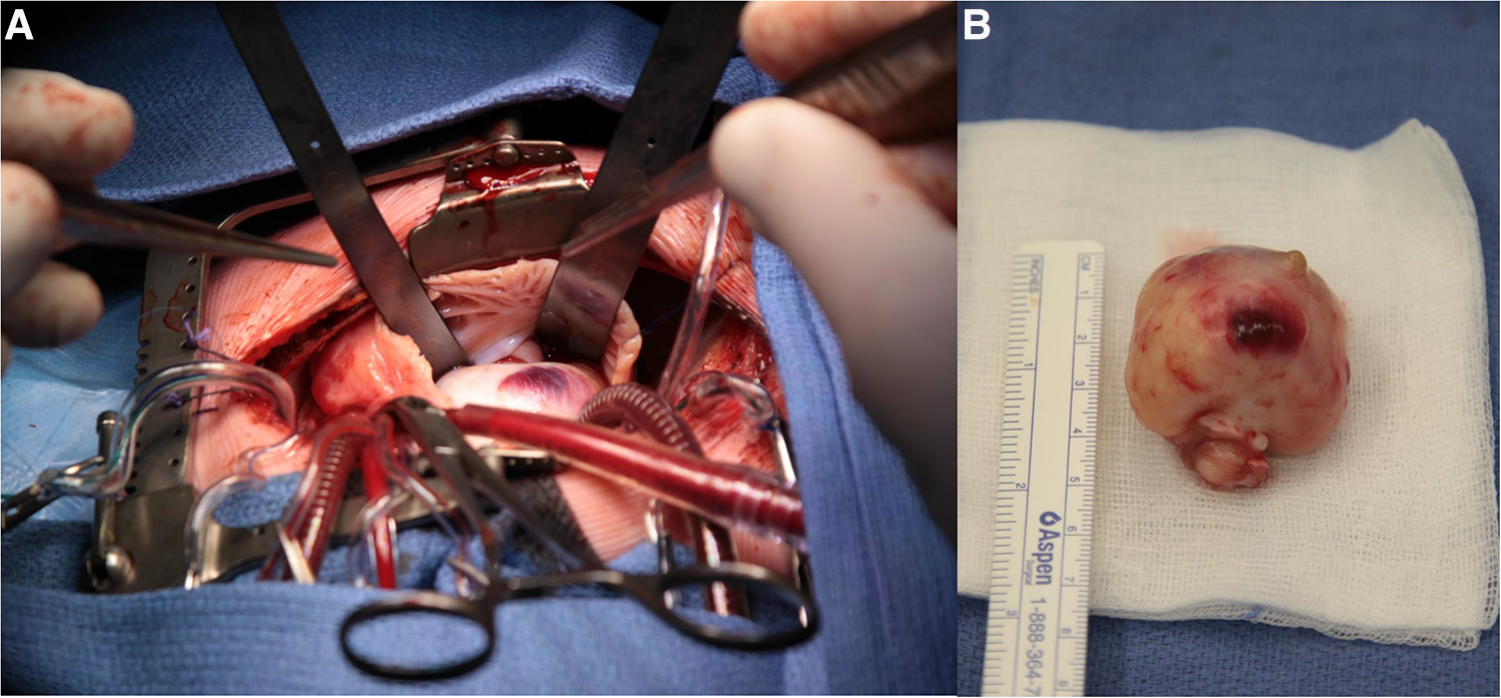
(**A**) Surgical view on cardiopulmonary bypass: right atrium opened with view on the right atrial appendix and the large mass bulging into the right atrium. (**B**) macroscopic view of resected tumor.

Urgent surgical resection was needed within 24 h after admission. The tumor was steeled on the anterior papillary muscle of the tricuspid valve (Figure 2A,B). A complete resection could be performed and subsequent a De Vega plasty of the tricuspid valve was established. The post-operative course was uneventful and the patient could be discharged from hospital 4 days after surgery.

**Figure 3:**
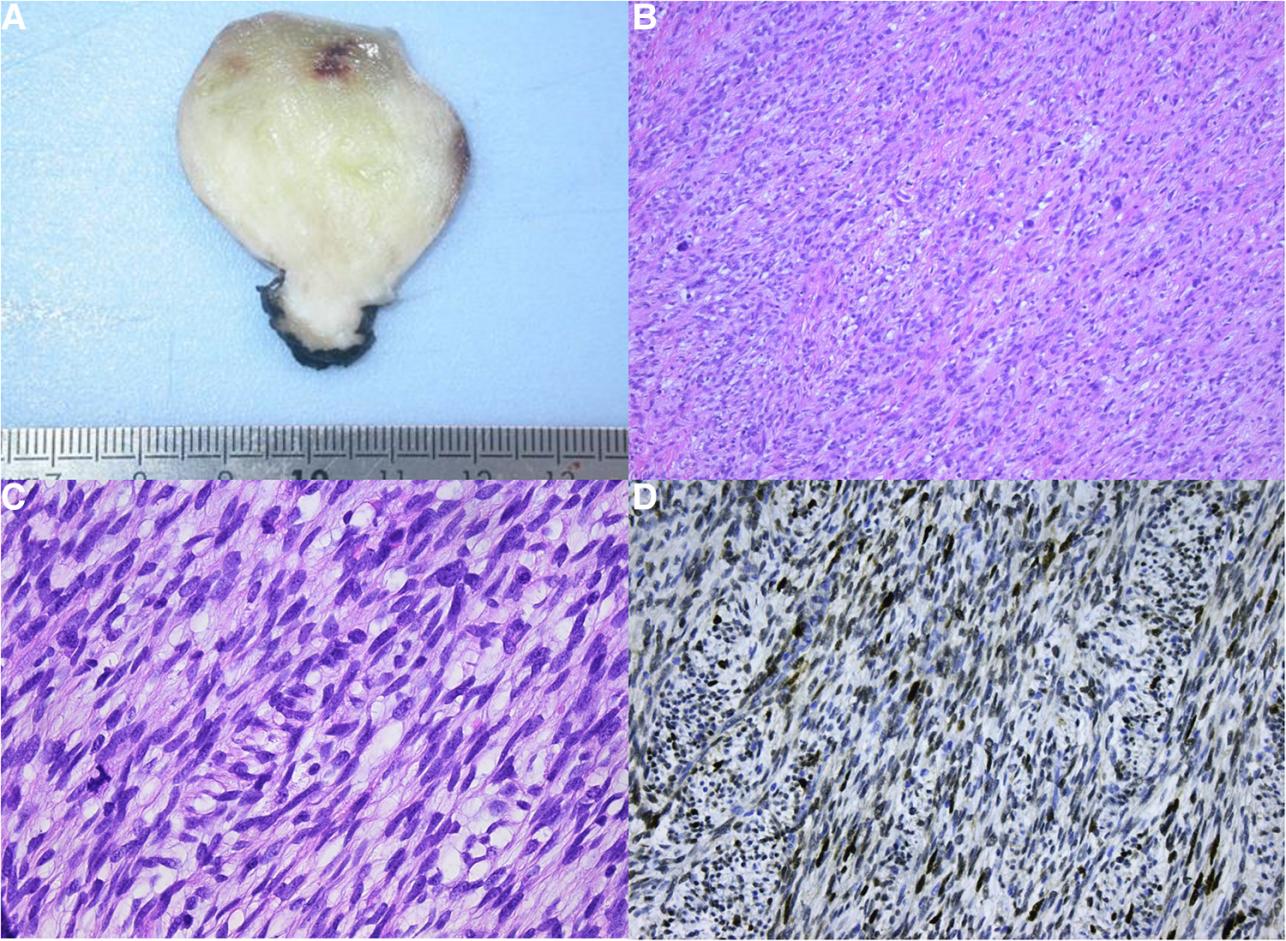
(**A**) Macroscopic view. (**B**) Low power view showing the fascicular arrangement of the tumor cells with prominent nuclear atypia. (**C**) At high power the nuclear atypia is appreciated to a better advantage. (**D**) Strong nuclear MDM2 expression.

Macroscopically the lesion was nodular and white with a diameter of 3.7〉× 3.5 × 3.5 cm (Figure 3A). After ﬁxation with 4% buffered formalin, samples were parafﬁn-embedded and 5-µm-thick sections were stained with haematoxylin and eosin (H&E). Light microscopy revealed a sharply demarcated lesion, not invading the residual myocardial tissue. The lesion consisted of spindle cells arranged in a storiform and herringbone pattern (Figure 3B). Less cellular, more fibrous and sometimes myxoid areas were admixed with cellular and atypical looking zones. Focally there was marked nuclear atypia with bizarre, giant nuclei (Figure 3C). The mitotic count ranged up to 5mitoses/10HPF. This not-specific morphology allows a very broad differential diagnosis. To narrow it down immunohistochemical stains were performed (Table 1). Strong expression of *MDM2* and *EGFR* was seen (Figure 3D). In addition, there was some expression of alfa-SMA neurofilament and EMA. The remaining stains were all negative.

**Table 1:** Immunohistochemical stains.

Antigen	Clone	Isotype	Dilution	Pre-treatment	Manufacturer
Alfa Smooth Muscle Actine (Alfa-SMA	1A4	IgG2a, kappa	RTU	high	DAKO IR611
Caldesmon	h-CD	IgG1, kappa	RTU	high	DAKO IR054
Calponin	CALP	IgG1, kappa	1:50	low	DAKO M3556
Chromogranin	LK2H10	IgG1, kappa	1:1,000	high	ThermoFisher Scientific
C-kit (CD117)	polyclonal	–	1:750	none	DAKO A4502
Desmine	D33	IgG1, kappa	RTU	high	DAKO code IR606
EGFR	E30 Dako EGFR25 Novocastra	IgG1, kappa IgG1	1:50 Dako 1:100 Novocastra	high	DAKO M7239 Novocastra NCL-L-EGFR-384
Epithelial membrane antigen (EMA)	E29	Ig2a, kappa	RTU	high	DAKO IR629
MDM2	IF2	IgG2b, kappa	1:50	high	Invitrogen 33–7,100
Myogenin	F5D	IgG1, kappa	RTU	high	DAKO IR067
Neurofilament	2F11	IgG1, kappa	RTU	high	DAKO IR607
S100	polyclonal	–	RTU	high	DAKO IR504
SOX10	polyclonal	–	1:1,500	high	Millipore AB5727
Synaptophysin	DAK-SYNAP	IgG1, kappa	RTU	high	DAKO IR660

FISH analysis was performed, and showed a gross amplification of *MDM2* gene in >50% of tumor cells. There was no amplification of *KIT*, *PDGFRA* or *EGFR* genes. A homozygous loss of *CDKN2A* on 9p21 was also present. As an intimal sarcoma is malignant, further tumor staging was performed. Blood work-up was unremarkable. Whole-body PET-CT-scan showed multiple hypermetabolic lymph nodes bilaterally in the retromandibular region, in the right supraclavicular and in the anterior part of the mediastinum. Bone scan using 99mTc-MDP as a tracer was unable to show skeletal metastases.

Adjuvant chemotherapy with doxorubicin (60 mg/m^2^ day1) and ifosfamide (3 g/m^2^ day1, 2 and 3) was proposed, but parents refused postoperative chemotherapy. Cardiac magnetic resonance imaging did not show any macroscopic neoplasm 6 months after surgical resection.

Follow-up was performed initially every 3 months the first year and yearly afterwards. To date 8 years after surgical resection echocardiography does show an unchanged small fibrotic zone at the base of the papillary muscle of the tricuspid valve, without tumor recurrence. There is a residual low grade of tricuspid valve regurgitation (1–2/4) and no stenosis.

## Discussion

Primary cardiac sarcomas are rare but represent the majority of primary malignant cardiac tumors. There are few series describing sarcomas of the heart, and the majority of publications are case reports (2, 5–10). Ramlawi et al. reports a single-institution experience on 95 cases over 25-year period and Isambert et al. on 124 cases in 33-year period of the French Sarcoma Group with a mean age of 43 and 47.1 years respectively (11, 12). Recently a large multi-institutional cohort of 747 patients with primary cardiac malignancies is reported from the US National Cancer Database (13). Among the reported sarcomas, angiosarcomas appear to be the most prominent type (32.3–40.4%) (5, 11–14). The most common sites for the sarcomas are the right (38.8–39%) and left atrium (33–37.2%), and far less frequent the right ventricle (5%–5.8%) (11, 12). Neuville et al. reports in a retrospective clinicopathologic and molecular analysis of one hundred primary sarcomas, intimal sarcoma as the predominant tumor type with occurrence rate of 42%, followed by angiosarcoma in only 26 of 100 cases (1). All but one angiosarcoma originated from the right heart, whereas 83% of intimal sarcomas were from the left heart. Most mentioned case series and case reports are findings in adult population, occurring mostly in fourth and fifth decade of life (7, 8, 15, 16). In children, the estimated incidence of all cardiac tumors, benign or malignant, is 0.0017%–0.027%, and primary malignancy is approximately 10% (13, 17). Primary cardiac malignancies have been studied in the SEER experience (4). A total of 25 pediatric patients (<20 years of age) were identified with primary cardiac tumor in a period between 1973 and 2008. Age-adjusted incidence is 0.00686 per 100,000 US population. Most common histology type was soft tissue sarcoma (40%), which was not further specified, followed by non-Hodgkin lymphoma and teratoma with the incidence of each of them being 12%.

Intimal sarcoma is a malignant mesenchymal tumor which is usually a poorly differentiated sarcoma composed of atypical spindle cells and/or pleomorphic cells with the possibility of myxoid areas or epithelioid morphology. Tumor cells generally exhibit immunoreactivity for vimentin and variable expression for smooth muscle actin but are usually negative for desmin and endothelial markers (18). In the study by Neuville et al. all cases showed overexpression and amplification of MDM2. More than two thirds were also positive for CDK4 and HMGA2, which has been confirmed in earlier reports (1, 18).

Primary cardiac sarcomas are highly aggressive tumors which rapidly infiltrate all the layers of the heart and metastasize rapidly, especially when right-sided (11). At the time of diagnosis up to 80% of patients have metastatic disease (19). Mean survival rates are poor and range from 3 months to 20 months (11, 12, 15, 20). The overall 5-year survival in the US National Cancer Database was 11.5% (13). Therapy is primarily surgical, with achievement of negative resection margins significantly prolonging patient survival (5, 11, 12, 15). Unfortunately complete tumor resection is only possible in less than 50% of patients. Adjuvant chemo- and/or radiation therapy remains controversial (13). Some groups suggest that these adjuvant therapies have no added value, while others suggest better survival rates in patients who had received chemotherapy and radiation therapy (5, 11, 12, 20).

The presented case is exceptionally rare for age of presentation as well as for tumor location for this type of tumor. According to the literature this is the first report of an intimal sarcoma within the ventricle in a young child. Previously a case of pulmonary artery intimal sarcoma has been reported in an infant and in the left atrium in another child (21, 22). Soft tissue sarcoma has been reported as the predominant tumor type in primary malignant pediatric cardiac tumors, though no further tumor type specification was mentioned. The location of the tumor in the presented case is rare as 83% of intimal sarcomas arise from the left heart (1, 4). On the other hand, intimal sarcoma is more often reported as a tumor of the large vessels, especially pulmonary artery and thus in some way arising from the right heart. Given the disproportion between reported cases of cardiac intimal sarcoma and the retrospective finding of intimal sarcoma as the predominant primary cardiac sarcoma in adults, it might be that intimal sarcoma of the heart in children is more prevalent than is assumed, however still extremely rare (1, 23). In cases of (pediatric) sarcoma of the heart it is important to search for amplification by FISH at least for *MDM2* (22, 24). In our case morphology and immune histochemistry results suggested the presence of intimal sarcoma. FISH analysis with amplification of *MDM2*, as well as loss of *CDKN2A*, supported the diagnosis.

In the case reported here complete resection of tumor could be obtained. Further tumor staging was negative apart from presence of multiple hypermetabolic lymph nodes on PET-CT scan. The lymphadenopathy could happen due to multiple reasons, as inflammation, a recent infection or more likely due to congestion caused by the sarcoma blocking the flow in the right ventricle and causing the effusions in the pericardium and the pleura, as well as causing the ascites. In the diagnostic work-up of intimal sarcomas CT and MRI are optimal for assessing tissue characteristics, infiltration and metastases (25). Given the high probability of occult dissemination at the time of diagnosis in this type of malignancy adjuvant chemotherapy was proposed, which was refused by the parents. Close follow-up is performed and with disease-free survival up to currently 8 years after surgical resection.

## Conclusion

Intimal sarcomas, undifferentiated mesenchymal tumors arising from the intimal layer of the ventricle are an extremely rare entity in children. The molecular diagnosis is confirmed by a *MDM2* amplification. Although the prognosis of primary cardiac sarcomas is overall poor, the presented case did show a good survival up to 8 years after surgical resection of the tumor.

## Data Availability

The original contributions presented in the study are included in the article/Supplementary Material, further inquiries can be directed to the corresponding author.
